# Age-, tumor-, and metastatic tissue-associated DNA hypermethylation of a T-box brain 1 locus in human kidney tissue

**DOI:** 10.1186/s13148-020-0823-x

**Published:** 2020-02-18

**Authors:** Jürgen Serth, Inga Peters, Natalia Dubrowinskaja, Christel Reese, Knut Albrecht, Michael Klintschar, Marcel Lafos, Alexander Grote, Albert Becker, Jörg Hennenlotter, Arnulf Stenzl, Hossein Tezval, Markus A. Kuczyk

**Affiliations:** 1 Klinik für Urologie und urologische Onkologie, Medizinische Hochschule Hannover, Carl-Neuberg-Str.1, D-30625 Hannover, Germany; 2 Brandenburgisches Landesinstitut für Rechtsmedizin, Lindstedter Chaussee 6, D-14469 Potsdam, Germany; 3 Institut für Rechtsmedizin, Medizinische Hochschule Hannover, Carl-Neuberg-Str.1, D-30625 Hannover, Germany; 4 Institut für Pathologie, Medizinische Hochschule Hannover, Carl-Neuberg-Str.1, D-30625 Hannover, Germany; 5 Evangelisches Klinikum Bethel, Klinik für Neurochirurgie, Burgsteig 13, D-33617 Bielefeld, Germany; 6 Institut für Neuropathologie, Universitätsklinikum Bonn, Sigmund Freud Str. 25, D-53105 Bonn, Germany; 7 Universitätsklinikum Tübingen, Klinik für Urologie, Hoppe-Seyler-Straße 3, 72076 Tübingen, Germany

**Keywords:** DNA methylation, Kidney tissue, Metastatic tissue, Metastasis, Age, Tumor progression, DNA hypermethylation

## Abstract

**Background:**

While a considerable number of tumor-specific hypermethylated loci have been identified in renal cell cancer (RCC), DNA methylation of loci showing successive increases in normal, tumoral, and metastatic tissues could point to genes with high relevance both for the process of tumor development and progression. Here, we report that DNA methylation of a locus in a genomic region corresponding to the 3′UTR of the transcription factor T-box brain 1 (*TBR1*) mRNA accumulates in normal renal tissues with age and possibly increased body mass index. Moreover, a further tissue-specific increase of methylation was observed for tumor and metastatic tissue samples.

**Results:**

Biometric analyses of the TCGA KIRC methylation data revealed candidate loci for age-dependent and tumor-specific DNA methylation within the last exon and in a genomic region corresponding to the 3′UTR *TBR1* mRNA. To evaluate whether methylation of *TBR1* shows association with RCC carcinogenesis, we measured 15 tumor cell lines and 907 renal tissue samples including 355 normal tissues, 175 tissue pairs of normal tumor adjacent and corresponding tumor tissue as well 202 metastatic tissues samples of lung, bone, and brain metastases by the use of pyrosequencing. Statistical evaluation demonstrated age-dependent methylation in normal tissue (*R* = 0.72, *p* < 2 × 10^−16^), association with adiposity (*P* = 0.019) and tumor-specific hypermethylation (*P* = 6.1 × 10^−19^) for RCC tissues. Comparison of tumor and metastatic tissues revealed higher methylation in renal cancer metastases (*P* = 2.65 × 10^−6^).

**Conclusions:**

Our analyses provide statistical evidence of association between methylation of *TBR1* and RCC development and disease progression.

## Background

Incidence of kidney and renal pelvis malignancies including renal cell cancers (RCC) have been reported to be among the top ten of newly diagnosed human cancers showing a sixth and tenth rank for males and females, respectively, in the USA [1]. While localized RCC demonstrates 5-year survival rates of more than 90%, metastasized tumors, detected in about 25% of primary RCC diagnoses, are still associated with a poor median overall survival of about 9 to 20 months [2, 3]. Previously, considerable progress has been made in the comprehensive molecular description of the clear cell variant of RCC (ccRCC) by The Cancer Genome Atlas network (TCGA) study using the kidney renal clear cell carcinoma (KIRC) tissue and data collection [4]. As a result of the KIRC study branch, it now appears debatable whether mutational analyses of tumors may be of a direct translational clinical use, considering that almost every of the tumors subjected to the study turned out to exhibit an individual mutation profile [4]. In contrast, numerous studies before as well as the KIRC study itself showed that DNA methylation occur with high frequency in RCC. Genes undergoing DNA methylation and epigenetic silencing of expression frequently show loss of function and can as a result match the classical mechanism of tumor suppressor inactivation due to genetic alterations [5–7].

DNA methylation in normal kidney tissues has been found often to be associated with age and other epidemiologic risk factors [8–10] as well as renal cell cancer risk [11]. Moreover, studies found that DNA methylation can be linked to characteristics of RCC or ccRCC such as histology [12], clinical stage [13], histological grade [13], state of local or distant metastasis [13, 14], prognosis of overall survival or time of disease recurrence [6, 7, 11, 15–18] as well as prediction of therapy response [18, 19]. Hence, the search for new epigenetic marks such as DNA methylation associating with crucial steps of carcinogenesis may provide a rationale for the development of useful clinical biomarkers, starting points for subsequent targeted functional analyses as well as epigenetic-based therapies [20]. Thus biometrical analysis of genome-wide data such as from the TCGA KIRC study providing methylation information for approx. 435,000 loci in ccRCC and also normal kidney tissues substantially improves the efficacy of the search for new epigenetic candidate markers of potential use for the clinical management of ccRCC.

As a result of in silico analysis of TCGA KIRC data for age-related DNA methylation, a methylated candidate loci located in a genomic region corresponding to the 3′UTR of the T-box brain 1 (*TBR1*) mRNA was identified. TBR1 is a T-box transcription factor and is involved in brain development and neural migration. Neurological disorders have been attributed to homozygous as well heterozygous loss of *TBR1* function due to mutations and chromosomal deletions [21]. In contrast, only two studies describe at yet association of genetic alterations and malignant diseases [22, 23]. Moreover, DNA-methylation analyses including a *TBR1* locus used in the present study have been previously described for methylation-based age prediction from normal cells in saliva in a forensic context [24].

Here, we describe the measurement of relative methylation of one *TBR1* locus amenable to pyrosequencing analysis within the region of interest revealing that methylation accumulates with age, associates with adiposity in normal tissues, shows tumor-specific hypermethylation and show maintenance and/or enrichment in RCC derived metastatic tissues.

## Results

### In silico identification of age-related methylated loci using KIRC

**Fig. 1:**
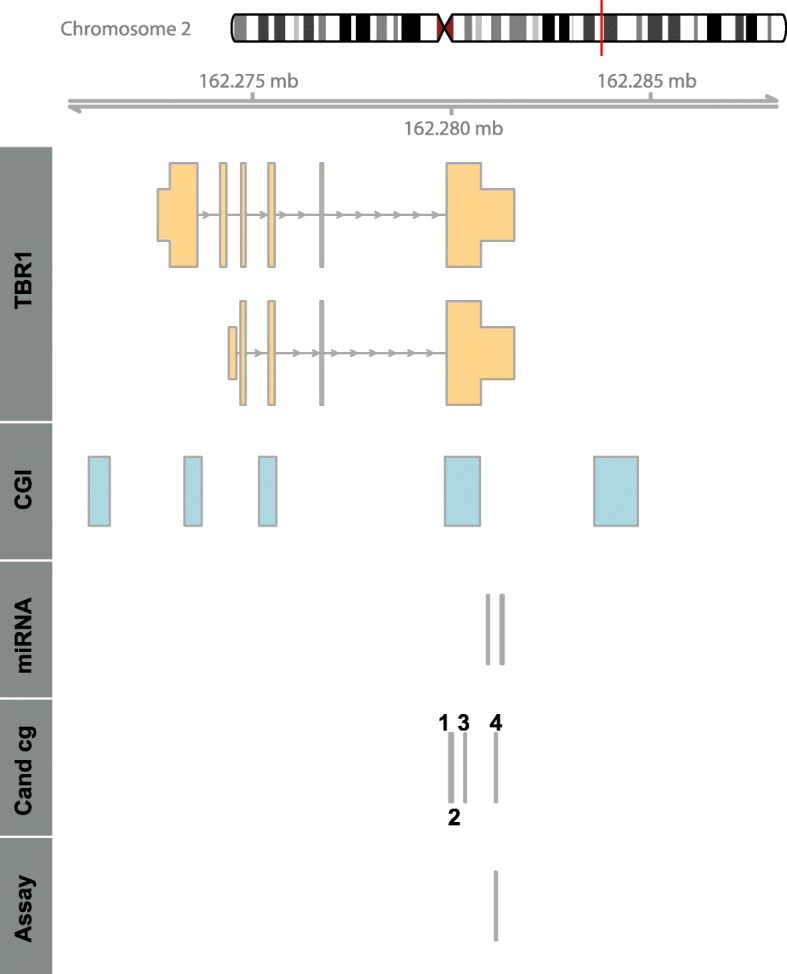
Genomic organization of the *TBR1* gene on chromosome 2 showing two transcripts: exons and genomic regions corresponding to the 5′- and 3′UTRs of *TBR1* mRNA are presented with thick and thinner boxes, respectively, distribution of CpG islands (CGI), putative target binding sites of microRNAs on corresponding *TBR1* mRNA, location of candidate CpG sites (C and cg:), and location of the pyrosequencing assay (Assay) are shown. Numbers refer to the candidate CpG sites cg05301866 (1), cg06942701 (2), cg06488443 (3), and cg12757011 (4)

KIRC methylation and age data of 175 tumor adjacent normal renal tissues were subjected to Pearson correlation analysis and ranked using the obtained coefficients of correlation as a measure. Among the top 50 CpG sites identified as candidates for age-related methylation in the genome-wide biometric analysis, we identified four CpG loci annotating either to the last exon or the genomic region corresponding to the 3′UTR of the *TBR1* mRNA (Fig. 1): cg05301866, cg12757011, cg06942701, and cg06488443. All candidate sites demonstrated Bonferroni-Hochberg adjusted *P* values of less than 1 × 10^−10^ and coefficients of correlation of *R* = 0.689–0.648 compared to a range of coefficients of correlation from *R* = 0.763 (first rank) to *R* = 0.646 (50th rank) for the top 50 candidate loci.

To narrow down the group of candidates a review of literature for known or supposed associations between gene alterations and cancer was carried out revealing the *TBR1* gene as a candidate gene. Technical evaluation of candidate CpG loci identified the cg12757011 locus (chr2q24.2: pos. 162,281,112) as a favorite target for pyrosequencing analysis. UCSC table browser in silico analysis shows that the cg12757011 locus is located in a genomic region corresponding to the 3′UTR of the *TBR1* mRNA. This section also includes microRNA response elements of the putatively cancer-related microRNAs miR-19, -375, -27ab, and -141/200a [25]. Genomic locations of *TBR1*-associated CpG islands (CGIs), the candidate CpG sites, the pyrosequencing assay and microRNA response elements (Fig. 1) as well as exemplary primary data for pyrosequencing analysis (Additional file 1: Figure S1) are presented.

### *TBR1* methylation frequently occurs in urological cancer cell line tumor models

**Fig. 2:**
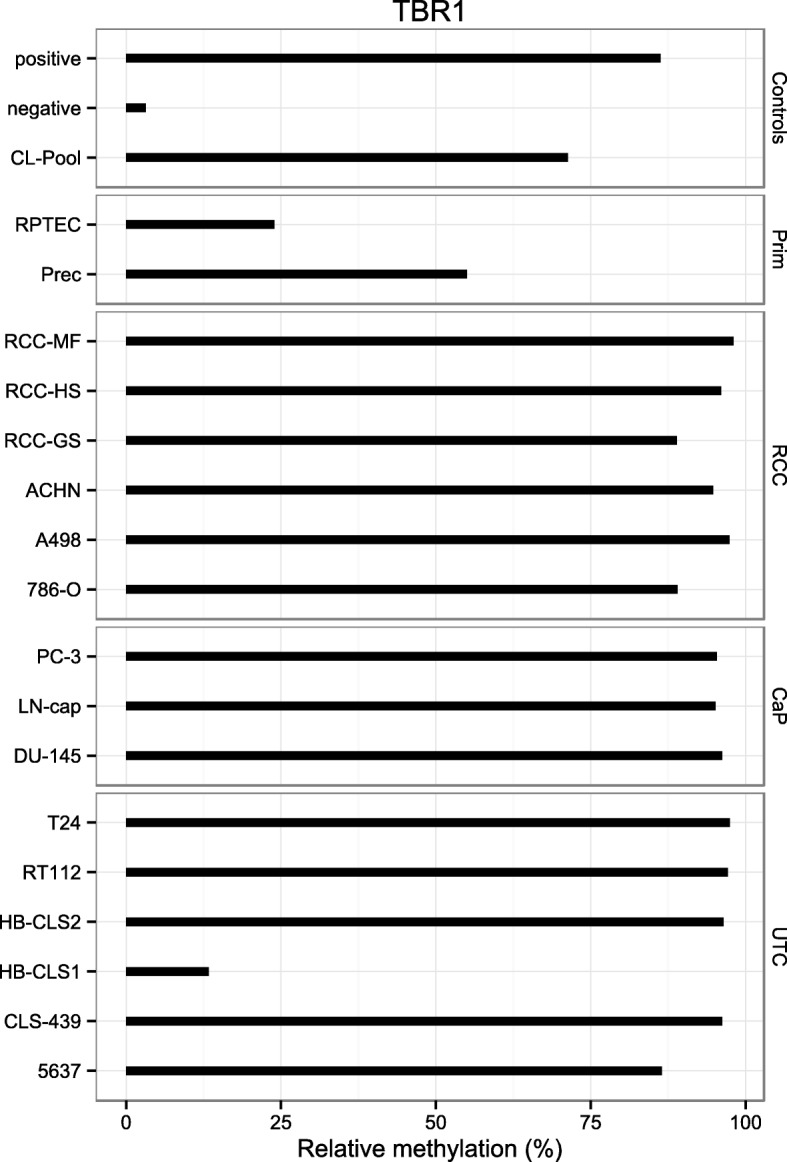
Relative *TBR1* methylation levels in controls (positive, negative, and cell line pool controls), in normal epithelial primary cells of kidney (RPTEC, Prec) and cancer cell lines of renal cell cancer (RCC), cancer of the prostate (CaP), and urothelial cancers (UTC)

Relative methylation in cell lines as measured by pyrosequencing demonstrated high level methylation of the *TBR1* locus in nearly all of the human cancer cell line models (Fig. 2). Six of six (100%), three of three (100%), and five of six (83%) of tumor cell lines for renal, prostate, and urothelial cancers revealed relative methylation levels of greater than 80%. Normal primary epithelial cells from kidney and prostate as well as the HB-CLS1 human bladder cancer cell line were detected with relative methylation values of 25–50%.

### *TBR1* DNA methylation in normal autopsy tissue shows association with age and adiposity

**Fig. 3:**
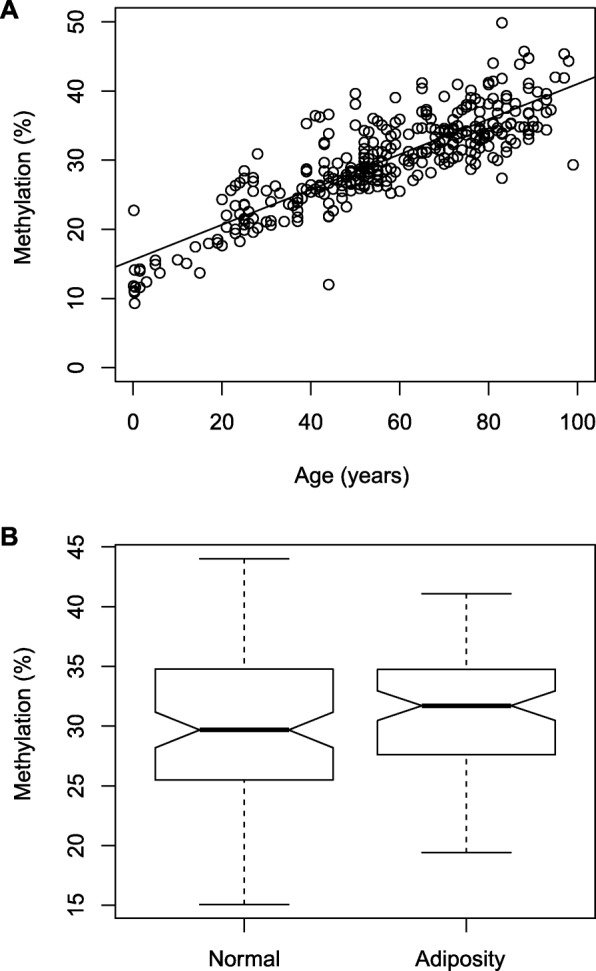
**a** Measurement of *TBR1* methylation in normal autopsy tissue samples and correlation with age of tissue donators. A strong correlation between relative methylation levels and age was found (*R* = 0.849, *P* < 2 × 10^−16^) showing a slope of 0.25% methylation increase per year. **b** Box plot comparison of relative TBR methylation levels measured for the normal and adiposity tissue group. Notches indicate estimated confidence intervals for group medians

Methylation analysis of 355 normal autopsy renal tissue samples revealed a strong correlation of relative methylation with age of tissue donators (*R* = 0.849, *P* < 2 × 10^−16^). Hence statistically viewed, over 70% of the observed variance in methylation can be explained by the variable age. The regression plot shows that methylation accumulates with age exhibiting an increase of 0.25% methylation per year (Fig. 3a). Noteworthy, increased methylation is observed early as even tissues aged between 0 and 5 years demonstrated relative methylation in the range of 10–15%. We also investigated whether relative methylation in autopsy tissue specimens shows association with body mass classification for obesity of tissue donators and compared subsets of 98 samples of normal (BMI 20–25) vs. 84 samples of the adiposity group (BMI > 30). Using bivariate logistic regression analysis and age as a covariate to account for age-related effects (Fig. 3b), we found a slight but statistically significant increase in *TBR1* methylation for the obesity subgroup (31.6% mean methylation) in comparison to the normal weight group (30.1% mean methylation) exhibiting only *TBR1* methylation but not age as a significant variable (*P* = 0.019, OR = 1.11, 95%CI 1.02–1.20) in the bivariate regression model. No significant difference could be detected for tissue specimens obtained from female and male donators (*P* = 0.31, OR = 0.97, 95% CI 0.91–1.03)

### The *TBR1* candidate locus is hypermethylated in renal cell cancer

**Fig. 4:**
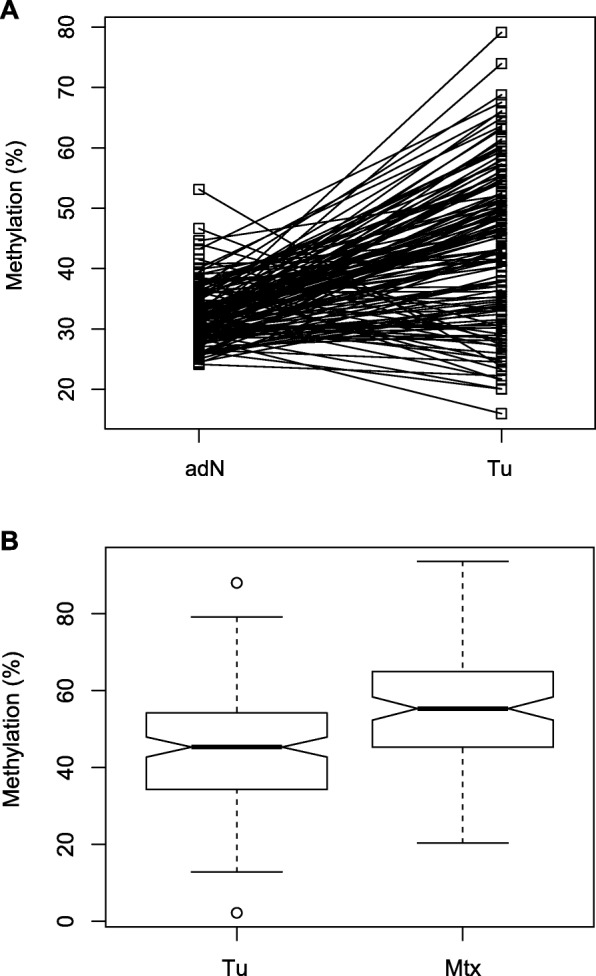
**a** Comparison of paired adjacent normal (and) and tumor (Tu) tissue methylation. Lines connect paired tissue samples. Statistical comparison of paired methylation values was carried out by use of the paired *t* test and showed significant hypermethylation for tumor tissues (paired two-sided *t* test, *p* = 6.1 × 10^−19^). **b** Box plot comparison of relative *TBR1* methylation levels observed in RCC sample tissues (Tu) and independent metastatic tissue samples (Mtx). Note that in case of multiple metastatic tissue samples mean methylation values were presented in the Mtx group. Notches indicate estimated confidence intervals for group medians

To analyze whether the *TBR1* candidate locus shows tumor-specific hypermethylation, we measured 175 corresponding tissue pairs of tumor adjacent normal and tumor tissue specimens (Fig. 4a). Paired tissue analysis revealed that by far the largest part of tumor tissues shows hypermethylation in tumor samples, a subset of corresponding tissue pairs did not exhibit higher methylation in tumors and a small part is characterized by hypo-methylated tumors, showing overall clear hypermethylation in tumor tissues (paired two-sided *t* test, *P* = 6.1 × 10^−19^).

### DNA methylation of *TBR1* locus increases in tissues of renal cancer metastases

We further compared methylation of the *TBR1* locus in a subset of 135 primary tumor tissues clinically diagnosed to be free both of lymph node and distant metastasis and 202 cancer metastatic tissues isolated from 105 renal cell cancer patients suffering from metastatic disease. Metastases investigated for *TBR1* methylation originated in 142 (70%) cases from lung, brain, bone or lymph nodes (Table 3). Mean methylation values were calculated in case that two or more metastatic tissues were measured per patient and compared to the independent group of primary cancers clinically classified as negative for lymph node or distant metastasis. We found that methylation in renal metastatic tissues increased by 5.2% to a mean value of 49.4% in comparison to a mean methylation of 44.1% in primary cancer tissues (Fig. 4b, logistic regression: *P* = 0.009). Review of relative methylation levels in in renal metastases revealed in part substantial variation within multiple metastases isolated from a single patient (data not shown).

### Association of *TBR1* methylation with clinico-pathological parameters

**Fig. 5:**
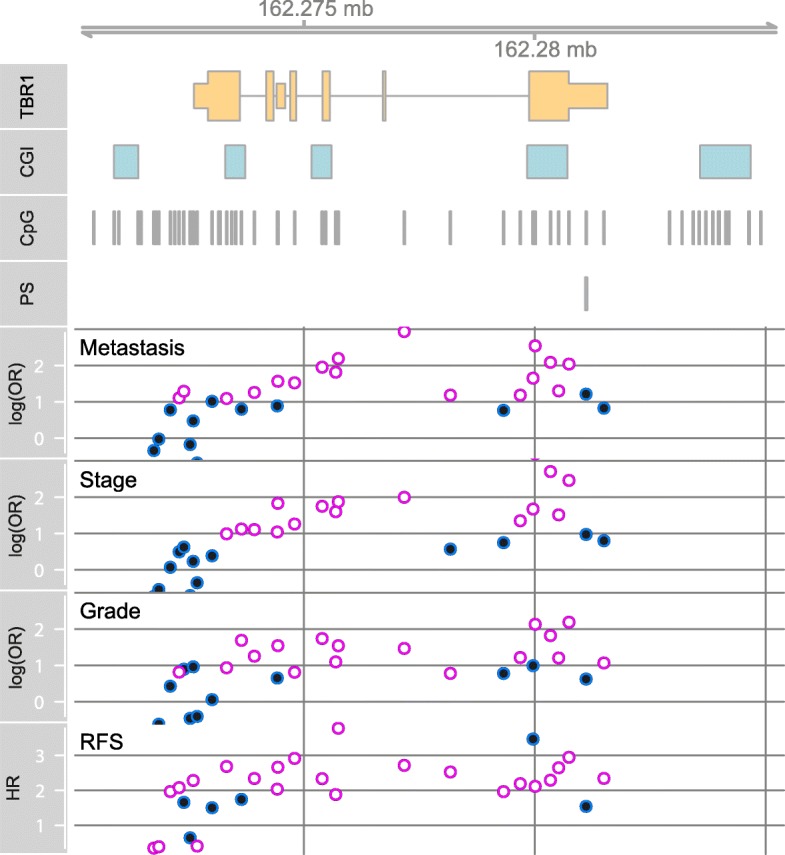
In silico analysis of association of CpG site methylation as reported by the TCGA-KIRC analysis with clinico-pathological parameters as well as recurrence-free survival (RFS) of patients in context with aggregated genomic organization of the *TBR1* gene (TBR1), localization of CpG islands (CGI), annotated CpG sites (CpG), and pyrosequencing assay (PS). Plots show the logarithm of odds ratios (log(OR)) obtained in bivariate logistic regression analyses for association with state of metastasis, high-stage (T3 or T4) tumors, and high-grade (G2–3, G3 ) tumors for each CpG site amenable to statistical evaluation. Open circles indicate analyses exhibiting statistical significance (*P* < 0.01) following Bonferroni-Hochberg adjusting for multiple testing. Filled circles indicate that statistical significance has not been reached (*P* > 0.01). Recurrence-free survival analysis is presented by hazard ratios (HR) obtained in univariate Cox-regression analysis without adjustment for multiple testing

Statistical evaluation of the candidate *TBR1* locus using the evaluation tissue cohort of primary tumors did not reveal any significant relationship in bivariate logistic regression analyses for the group of all RCC or ccRCC when including age as covariate for the clinic-pathological parameters of clinical state of distant (*P* = 0.27 and *P* = 0.70 for all RCC and ccRCC groups) and lymph node (*P* = 0.44 and *P* = 0.53) metastasis, high (> = G2–3) vs. low (< = G2) histological grade (*P* = 0.12 and *P* = 0.34) and high (> = T3) vs. low (< = T2) clinical stage (*P* = 0.21 and *P* = 0.12). Considering that the candidate CpG site cg12757011 was identified by biometric analysis primarily aiming at detection of age-related DNA methylation rather than association with clinical parameters we asked whether other CpG sites in the *TBR1* gene may show statistical association with adverse clinic-pathological parameters. In silico analysis using the TCGA KIRC data of 284 tumor patients then revealed that several CpG sites located in the gene body region upstream of the candidate locus show significant association with adverse clinic-pathological parameters as well as recurrence-free survival of patients (Fig. 5). So, presumably one larger group of neighbored CpG sites located between exons 3 and 6 demonstrated comparatively higher odds ratios for association with state of distant metastasis, high-stage tumors, and loss of differentiation in tumors in bivariate logistic regression analyses including age as a covariate. Correspondingly, these loci exhibited also comparatively increased hazard ratios in cox regression analyses indicating that loci statistically associated with adverse clinico-pathologic parameters were also related with shortened recurrence-free survival of patients (Fig. 5, Additional file 2: Table S1).

### Loss of *TBR1* mRNA expression

In silico comparison of mRNA expression using the KIRC data demonstrated an approx. 2.3-fold reduction of expression in tumor tissues compared to normal tissues (*P* = 0.012, paired *t* test).

## Discussion

Identification of new methylation marks associated with tumor risk and/or progression in specific human tissues may provide the base of molecular biomarkers for diagnosis and individualized therapy approaches as well as give reasonable starting points for functional analyses of kidney cancer tumor biology. In view that approx. 80% of cancer-related methylated loci also show age-related methylation in normal tissues [26] in silico analyses of genome-wide methylation databases such as the TCGA KIRC data for renal tissues could also be a rationale for the identification of new epigenetic marks with possible relevance for renal cell cancer.

Our genome wide search in the KIRC data set identified four out of the 50 top ranking age-dependent methylated CpG sites in normal kidney tissues to be located in a genomic region corresponding to the last exon and the 3′UTR of the *TBR1* mRNA. Evaluation by use of the autopsy normal tissue sample cohort revealed full confirmation of the biometric discovery study result as the candidate *TBR1* CpG site demonstrated a strong and statistical robust linear relationship between DNA methylation and age of tissue donators.

Of note, comparison of normal and obesity-related tissue sample subsets suggested adiposity as an additional epidemiologic factor for higher *TBR1* methylation in normal renal tissues. However, differences in mean methylation values of compared groups seem to be limited and substantial overlap is observed in both distributions of methylation values. Interestingly, we previously observed a similar result for methylation of the RASSF1 gene in normal kidney cells also discussing the possible hypothetical explanations for these findings [10]. On the other hand, methylation analyses in normal kidney tissues revealed that methylation of the TBR1 locus was found to be statistically associated with two known epidemiological risk factors in normal renal tissues. From a technical point of view, therefore our results show that biometric analysis of the TCGA KIRC data may serve not only for statistical answering of tumor-related questions but is also suited in principal for the identification of normal tissue alteration such as age- and lifestyle-associated epigenetic marks. Previous experimental studies used a subset of 1500–2000 CpG sites corresponding to several hundred genes and showed that CGI-DNA methylation in human solid normal tissues in general is often associated with age and environmental factors [27, 28]. Our results indicate that the KIRC methylation data of nominally 435,000 CpG sites are also usable for tissue-specific and genome-wide search of age-related epigenetic marks in normal kidney tissue.

Interestingly, so far, only few genes have been quantitatively characterized for age-dependent methylation in solid normal human tissues therefore limiting possibilities of comparison. Previous work of us analyzed the association of *RASSF1* and *SFRP1* CGI methylation and age in normal kidney tissue [11, 29]. In comparison, the observed variance in the linear regression model was by far lowest for *TBR1* which might be due to the biometrical genome-wide pre-selection of candidates for highest coefficients of correlation with age. Moreover, the highest average rate of annual gain in methylation of about 0.25% was observed for *TBR1* compared to *RASSF1* (0.15%) and *SFRP1* (0.06%). Recently, a considerable number of loci, including the *TBR1*–cg12757011 locus, have been analyzed to establish methylation-based age prediction from cytological saliva samples [24]. While the increase in relative methylation levels seems to be in good concordance when comparing results obtained for the age interval between 20 and 60 years for normal saliva and renal cells, apparently the normal kidney cells revealed a much higher coefficient of correlation of *R* = 0.85 compared to *R* = 0.17 in normal saliva cells.

Identification of cg12757011 as an age-related *TBR1* methylation locus which is located outside of a CGI seems not meeting current hypotheses postulating age dependent intra CGI-hypermethylation while CpG loci outside of CGIs are rather expected to show hypomethylation [26, 27].

Whether methylation in age dependent methylated loci shows further increase in tumor tissues, aggressive tumors and eventually in metastatic tissues is a question of fundamental interest because the preservation or even stepwise enrichment of cells carrying the methylation mark, detected by maintenance or successive gains in relative methylation values, would suggest a contribution of the corresponding candidate gene to tumor development, tumor progression and metastasis [30]. Here, we found that both tumor and metastatic tissues show a specific increase in *TBR1* methylation when compared to the respective predecessor tissues. These results are in line with a gradually enrichment of cells carrying *TBR1* methylation during progression from normal to tumor tissues and tumor to metastatic tissues. Therefore, our results statistically support the view that *TBR1* methylation promotes the development both of tumor cells as well as of metastatic cells growing out of primary tumor tissues.

We also questioned whether tumor progression and a concurrent gain of *TBR1* methylation can be also detected within the group of primary tumors when comparing for the clinical status distant metastasis, stage and grade of tumors. However, corresponding statistical associations were not found in our cohort apparently contradicting the findings in metastatic tissues. On the other hand, two arguments probably put these findings into perspective. First, tumor heterogeneity for *TBR1* epi-alterations could impede the detection of metastasis-positive tissues leading to a decreased statistical power, in particular when considering that only 16% of primary tumors were clinically classified as metastasis positive. Second, our in silico analysis demonstrated that cg12757011 is located in the 3′ flank of a region of other CpG sites showing a much more pronounced association with adverse clinic-pathological parameters as well as worse survival of patients. Therefore we assume that adaption of study design by increasing the number of primary tumors with positive status of distant metastasis together with assessment of CpG sites located in the 5′ neighborhood could improve detection of metastasis in primary kidney tumors. Taking into account that our evaluation study using the TCGA KIRC data confirmed association of *TBR1* methylation with disadvantageous clinical and survival parameters our study approach comparing renal tissues from normal towards full metastatic tissue nevertheless might provide an efficient way for identification of new genes relevant for tumor development and progression.

*TBR1* alterations have been found primarily in neurological disorders thus far, while information about *TBR1* alterations in human cancers is sparse [21]. So, copy number variations for *TBR1* have been reported for glioblastomas [22] and mutation of the *TBR1* gene was found in medulloblastomas, an embryonic cancer of the brain [23]. In line with thin information about *TBR1* alterations occurring in tumors, methylation of the gene has not been reported until now in the context of any human cancer to the best of our knowledge. Therefore, our results give additional evidence that *TBR1* alterations play also a role in human cancers, in particular when considering that 11 out 12 cancer models representing the 3 urological tumor entities of kidney, urothelial, and prostate cancer show high *TBR1* methylation. As our study provided statistical evidence which is in line with a possible contribution of *TBR1* alterations in the development and progression of RCC, functional studies are required to clarify the causal relevance of TBR1 alterations for renal cancers.

Taking into account that our analysis of KIRC data also revealed a significant tumor-specific reduction of *TBR1* mRNA expression and biometrical data provided by the UCSC genome browser report the presence of microRNA response elements, from a theoretical point of view epigenetic alteration of both transcriptional as well as post-transcriptional control of TBR1 mRNA expression in RCC can be considered as possible starting points for functional analyses. So tumor-specific hypermethylation and loss of mRNA expression are a characteristic of epigenetic silencing of gene expression while the presence of microRNA response elements of the miR-200 family point to a possible alteration in regulation of cellular response to hypoxia, representing one of the most important pathophysiological processes in RCC development [20, 31].

## Conclusions

Our study identified a new epigenetic methylation mark in normal kidney tissue showing accumulation with age and further enrichment in tumor as well as metastatic kidney tissues, thus providing statistical evidence of association between TBR1 DNA methylation and RCC development and disease progression.

## Methods

### In silico analyses for candidate identification

For statistical analysis level 3 data of the TCGA KIRC HM450k methylation data set [4], the statistical software R 3.02 [32] and a x86 64 bit desktop computer platform with 32 GB RAM running under Windows 7 was used. Candidate age-dependent methylated loci were identified by Pearson correlation analyses of the normal tissue subset. Results were adjusted using Benjamini-Hochberg correction for multiple statistical testing.

### Primary cells and tumor cell lines

Renal proximal tubular epithelial cells (RPTEC) and normal primary prostatic cells (PreC) were purchased from Lonza (Basel, Switzerland) and renal, prostatic, and urothelial cancer cell lines RCC-MF,RCC-HS, RCC-GS, ACHN, A498, 786-O, PC-3,LN-cap, DU-145, T24, RT112, HB-CLS2, HB-CLS1, CLS439, and 5627 were obtained from cell line services (CLS, Eppelheim, Germany). Cells were grown according to the manufactures instructions presenting no more than 16 passages before DNA isolation.

### Study design, tissue donator, and patients’ characteristics

To evaluate the relevance of *TBR1* DNA methylation for RCC carcinogenesis, we measured four tissue cohorts including a total of 907 tissue samples. Three hundred and fifty-five fresh frozen normal renal tissue (No) samples from autopsies (Table 1) were analyzed in a cross-sectional study for the relationship of methylation and tumor risk factors age, sex, and body mass index (BMI). Tumor-specific hypermethylation was analyzed by comparison of 175 pairs of normal tumor adjacent (adN) and tumoral fresh frozen tissue (Tu) samples (Table 2). Methylation in renal metastases were analyzed in 202 RCC formalin-fixed paraffin-embedded (FFPE) samples from metastatic tissues of 105 patients isolated mostly from lung, bone, and brain (Table 3). TNM-classification and grading of tumors was carried out as described previously [18].

**Table 1:** Tissue characteristics of normal renal autopsy specimens

	All	^1^BMI-analysis
Donators		
Total	355	184
Age (years)		
Median (min-max)	57 ( 0.1–99)	57 (12–97)
Sex		
Female (%)	123 (34.6%)	67 (36.4%)
Male (%)	219 (61.7%)	116 (63.0%)
na (%)	13 (3.7%)	1(0.5%)
Body mass index		
Median (min-max)	26.1 (7–59)	24.6 (20–59)
na (%)	3 (0.9%)	0(0%)

*na* data is not available for category

^1^BMI analyses were carried out with a subset of donators classified as normal BMI (20–25) or adiposity (BMI > 30)

**Table 2:** Clinico-pathologic features of tumor patients

		All RCC	%	ccRCC	%
Total cases	No.	175	100	140	100
Histology					
	ccRCC	140	80	140	100
	papRCC	23	13.1	0	0
	Chrom. RCC	3	1.7	0	0
	Mixed histol.	5	2.9	0	0
	Other	4	2.3	0	0
Sex					
	Female	63	36	53	37.9
	Male	112	64	87	62.1
Age					
	Median	65		65	
	Min-max	35–91		35–90	
Metastasis					
	M0	143	81.7	113	80.7
	M+	29	16.6	24	17.1
	na	3	1.7	3	2.1
Lymph node met					
	N0	155	88.6	126	90
	N+	15	8.6	9	6.4
	na	5	2.9	5	3.6
T-classification					
	pT1	11	6.3	8	5.7
	pT1a	58	33.1	45	32.1
	pT1b	40	22.9	33	23.6
	pT2	8	4.6	7	5
	pT3	5	2.9	2	1.4
	pT3a	16	9.1	13	9.3
	pT3b	31	17.7	29	20.7
	pT3c	4	2.3	3	2.1
	pT4	1	0.6	0	0
	na	1	0.6	0	0
Differentiation					
	G1	34	19.4	30	21.4
	G1–2	18	10.3	11	7.9
	G2	96	54.9	77	55
	G2–3	9	5.1	5	3.6
	G3	18	10.3	17	12.1
Localized disease^1^		103	58.9	80	57.1
Advanced disease^1^		66	37.7	55	39.3
na		6	3.4	5	3.6
Paired samples	No.	175	100	140	100

*Abbreviations: ccRCC* clear cell renal cell carcinoma, *papRCC* papillary, *chrom.*chromophobe*, met.*metastasis*, na* not available

^1^Localized and advanced disease defined as pT< = 2,N0, M0, or pT > = 3 and/or N+, M+

**Table 3:** Characteristics of metastatic tissue samples

		*n*	%
Patients	No.	105	100
Tissue samples	No.	202	100
Age	Median	66.5 (41–86)	
Localization			
	Lung	55	27.2
	Brain	32	15.8
	Bone	24	11.9
	Lymph nodes	31	15.3
	Adrenal	18	8.9
	Muscle	7	3.5
	Other	35	17.3

### Nucleic acid extraction, DNA bisulfite conversion, and DNA methylation analysis

Histological analysis for tumor cell content in control sections, DNA isolation from frozen section, and punches of formalin-fixed paraffin-embedded tissue samples as well as bisulfite conversion of DNA were carried out as reported previously [17, 19]. DNA from cancer cell lines and primary cells was obtained using standard proteinase K digestion and phenol-chloroform extraction. Methylation analysis was carried out by pyrosequencing. Primers were designed by use of the PyroMark Assay Design 2.0 software (Qiagen, Hilden, Germany). Pyrosequencing was performed as described recently [Tezval, 2016]. Primer sequences used for PCR and pyrosequencing were each in 5′-3′ direction, GGTGGGTTTAGGTTTTAGAGT (forward), CTCCCCCTCCTCTTTCTCTTACCTCCT (reverse), and GAGTTAATATTTTATGGTTAATGTG (sequencing). The sequence to analyze was 5′-GAGGTYGAGA TTTGGYGGGT YGGAATYGTT GTTGTTTGAT AGGATTGTTT–3′. The CpG sites analyzed were located on chromosome 2 at positions 162,281,112, ~118, ~123, ~133 (UCSC, hg19 data) in a genomic region corresponding to the 3′UTR of *TBR1* mRNA (Fig. 1). PCR reactions and preparation of pyrosequencing templates were carried out as described before [33].

### Statistical analyses

For analysis of age-dependent methylation Pearson correlation analysis was applied. Comparison of methylation in tumor and paired tumor adjacent normal tissues were carried out using the two-sided paired *t* test. Sub group comparisons for association with obesity or clinico-pathological parameters were performed by the use of univariate and bivariate logistic regression models if necessary following dichotomization as specified. Comparison of metastatic tissue samples with independent primary cancer tissues were carried out following aggregation of multiple metastasis data using calculation of the mean metastatic methylation value per patient and the two-sided *t* test for independent sample groups. All statistical calculations were done using R 3.02 [32].

## Supplementary information

**Additional file 1: Figure S1.** Primary data showing pyrosequencing results obtained for normal kidney tissue samples of age 3 years (A), 91 years (B), paired tumor adjacent histopathological normal (C) and tumoral (D) tissue samples and a renal cancer brain metastasis tissue sample (E) exemplarily showing overall increase of methylation in renal tissues of different normal and malignant states.

**Additional file 2: Table S1.** Cox regression analysis of TCGA KIRC data for for association of TBR1 CpG methylation and recurrence-free survival of patients.

## Data Availability

The datasets generated and/or analyzed during the current study are not publicly available because of potentially possible impairment of data protection concerns of patients.
