# Supplementary figures and images for: Age-, tumor-, and metastatic tissue-associated DNA hypermethylation of a T-box brain 1 locus in human kidney tissue
Source: Clin Epigenetics. 2020 Feb 18;12:33. doi: 10.1186/s13148-020-0823-x (PMC7029553; doi:10.1186/s13148-020-0823-x)

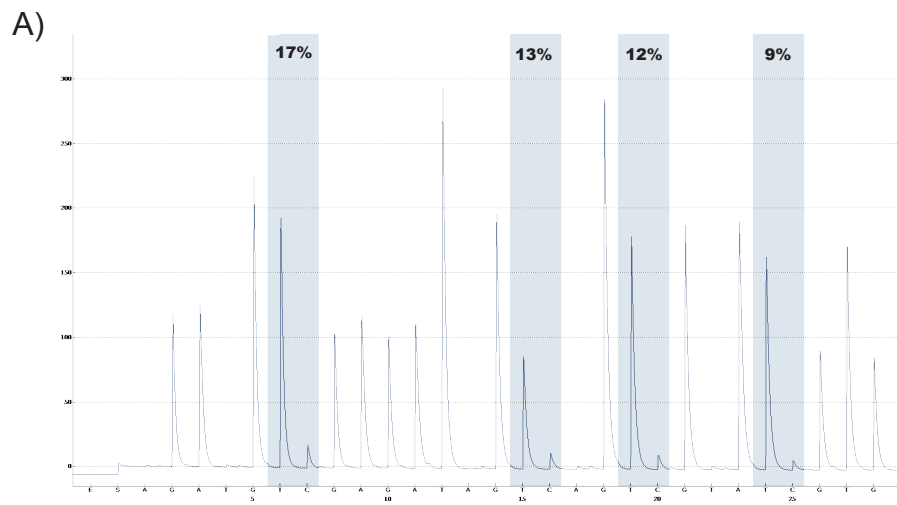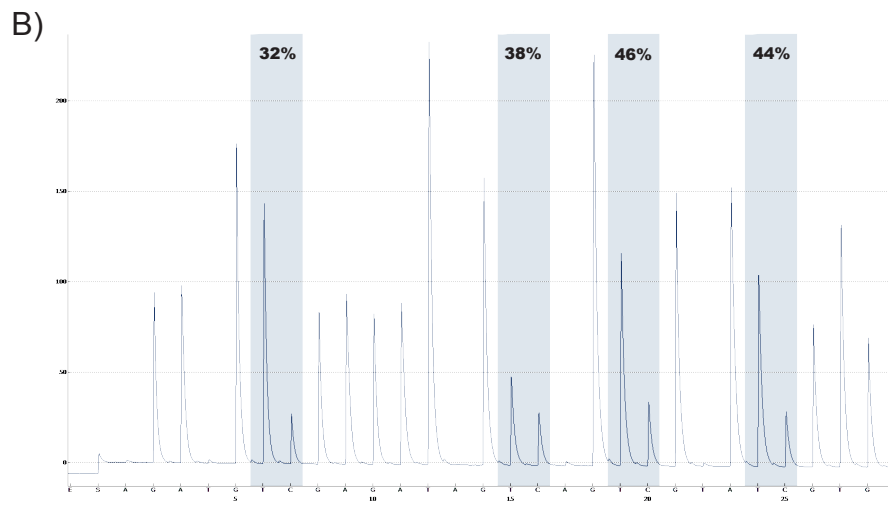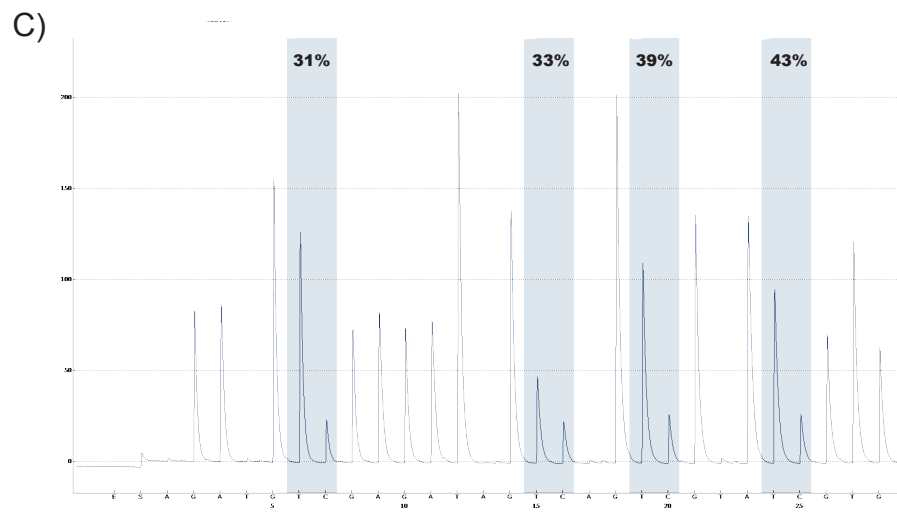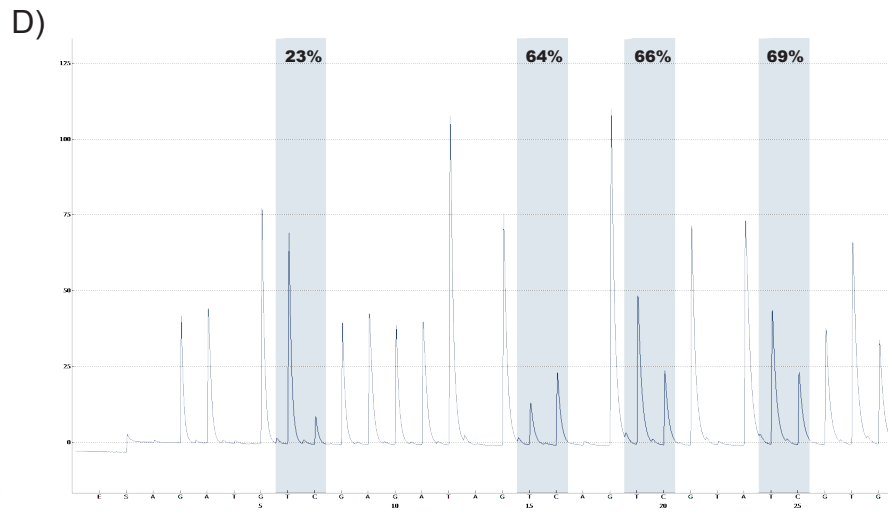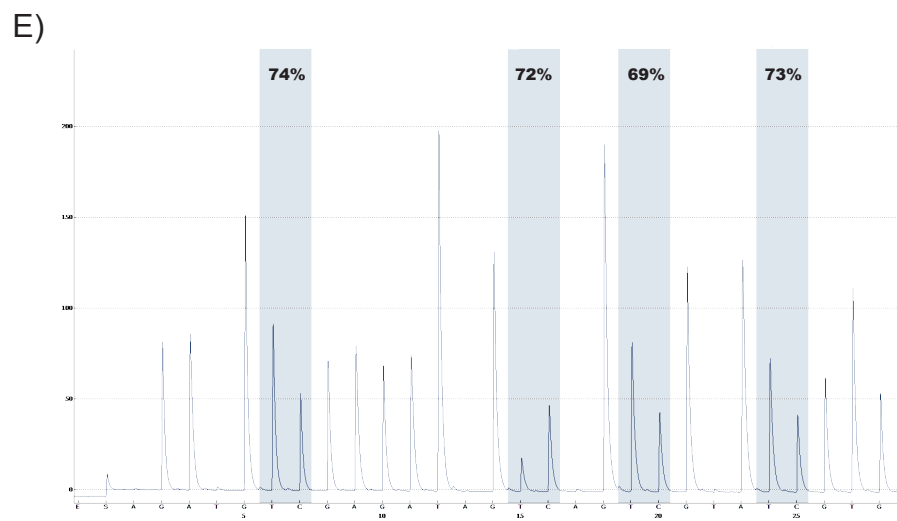

Supplement: Supplementary file 1 — Additional file 1: Figure S1. Primary data showing pyrosequencing results obtained for normal kidney tissue samples of age 3 years (A), 91 years (B), paired tumor adjacent histopathological normal (C) and tumoral (D) tissue samples and a renal cancer brain metastasis tissue sample (E) exemplarily showing overall increase of methylation in renal tissues of different normal and malignant states. [file 13148_2020_823_MOESM1_ESM.pdf]
