# Supplementary material for: Age-, tumor-, and metastatic tissue-associated DNA hypermethylation of a T-box brain 1 locus in human kidney tissue
Source: Clin Epigenetics. 2020 Feb 18;12:33. doi: 10.1186/s13148-020-0823-x (PMC7029553; doi:10.1186/s13148-020-0823-x)
Supplement: Supplementary file 2 — Additional file 2: Table S1. Cox regression analysis of TCGA KIRC data for for association of TBR1 CpG methylation and recurrence-free survival of patients. [file 13148_2020_823_MOESM2_ESM.docx]

**Additional file 2: Table S1** Cox regression analysis of TCGA KIRC data for for association of TBR1 CpG methylation and recurrence free survival of patients

| CpG site | Chrom. | Position | p-Value | HR | CI_L | CI_H |
| --- | --- | --- | --- | --- | --- | --- |
| cg07548213 | chr2 | 162271751 | 0.007072 | 0.35 | 0.16 | 0.75 |
| cg06747888 | chr2 | 162271859 | 0.003097 | 0.39 | 0.21 | 0.73 |
| cg09019329 | chr2 | 162272107 | 0.003192 | 1.96 | 1.25 | 3.08 |
| cg20994660 | chr2 | 162272299 | 0.001279 | 2.08 | 1.33 | 3.24 |
| cg10954251 | chr2 | 162272400 | 0.015783 | 1.65 | 1.10 | 2.49 |
| cg21431041 | chr2 | 162272536 | 0.182119 | 0.64 | 0.33 | 1.23 |
| cg04004632 | chr2 | 162272604 | 0.002259 | 2.28 | 1.34 | 3.87 |
| cg14187678 | chr2 | 162272688 | 0.009794 | 0.40 | 0.20 | 0.80 |
| cg10395317 | chr2 | 162273011 | 0.049751 | 1.50 | 1.00 | 2.25 |
| cg26210445 | chr2 | 162273326 | 0.000084 | 2.68 | 1.64 | 4.38 |
| cg22743761 | chr2 | 162273648 | 0.159155 | 1.74 | 0.80 | 3.76 |
| cg14416523 | chr2 | 162273928 | 0.000116 | 2.34 | 1.52 | 3.60 |
| cg02767242 | chr2 | 162274424 | 0.000802 | 2.03 | 1.34 | 3.08 |
| cg07419021 | chr2 | 162274436 | 0.000007 | 2.66 | 1.74 | 4.07 |
| cg06148480 | chr2 | 162274799 | 0.001436 | 2.91 | 1.51 | 5.62 |
| cg00462525 | chr2 | 162275396 | 0.000557 | 2.33 | 1.44 | 3.77 |
| cg10997718 | chr2 | 162275690 | 0.002433 | 1.88 | 1.25 | 2.82 |
| cg00018128 | chr2 | 162275746 | 0.000000 | 3.78 | 2.37 | 6.01 |
| cg10479234 | chr2 | 162277174 | 0.000004 | 2.71 | 1.78 | 4.14 |
| cg08314300 | chr2 | 162278173 | 0.001063 | 2.52 | 1.45 | 4.39 |
| cg15837233 | chr2 | 162279324 | 0.001497 | 1.96 | 1.29 | 2.97 |
| cg14557487 | chr2 | 162279686 | 0.000927 | 2.19 | 1.38 | 3.48 |
| cg05301866 | chr2 | 162279964 | 0.014991 | 3.47 | 1.27 | 9.44 |
| cg06942701 | chr2 | 162280009 | 0.000573 | 2.11 | 1.38 | 3.23 |
| cg06488443 | chr2 | 162280341 | 0.000691 | 2.29 | 1.42 | 3.69 |
| cg06382344 | chr2 | 162280519 | 0.000003 | 2.64 | 1.76 | 3.97 |
| cg12520549 | chr2 | 162280741 | 0.000001 | 2.94 | 1.90 | 4.56 |
| cg12757011 | chr2 | 162281111 | 0.053514 | 1.54 | 0.99 | 2.38 |
| cg10942521 | chr2 | 162281498 | 0.000616 | 2.34 | 1.44 | 3.81 |

*Results of univariate Cox regression analysis are presented for 29 CpG sites annotated to the TBR1 gene. Location information (cg-notation, chromosome and position) , p-Values, hazard ratios (HR) and the lower (CI_L) and upper (CI_H) 95% confidence intervals are shown.*
